# Estimating confidence intervals in predicted responses for oscillatory biological models

**DOI:** 10.1186/1752-0509-7-71

**Published:** 2013-07-29

**Authors:** Peter C St John, Francis J Doyle

**Affiliations:** 1 Department of Chemical Engineering, University of California, Santa Barbara, CA 93106-5080, USA

**Keywords:** Bootstrap, Identifiability, Oscillatory models, Circadian rhythms, Sensitivity analysis, Parameter estimation

## Abstract

**Background:**

The dynamics of gene regulation play a crucial role in a cellular control: allowing the cell to express the right proteins to meet changing needs. Some needs, such as correctly anticipating the day-night cycle, require complicated oscillatory features. In the analysis of gene regulatory networks, mathematical models are frequently used to understand how a network’s structure enables it to respond appropriately to external inputs. These models typically consist of a set of ordinary differential equations, describing a network of biochemical reactions, and unknown kinetic parameters, chosen such that the model best captures experimental data. However, since a model’s parameter values are uncertain, and since dynamic responses to inputs are highly parameter-dependent, it is difficult to assess the confidence associated with these in silico predictions. In particular, models with complex dynamics - such as oscillations - must be fit with computationally expensive global optimization routines, and cannot take advantage of existing measures of identifiability. Despite their difficulty to model mathematically, limit cycle oscillations play a key role in many biological processes, including cell cycling, metabolism, neuron firing, and circadian rhythms.

**Results:**

In this study, we employ an efficient parameter estimation technique to enable a bootstrap uncertainty analysis for limit cycle models. Since the primary role of systems biology models is the insight they provide on responses to rate perturbations, we extend our uncertainty analysis to include first order sensitivity coefficients. Using a literature model of circadian rhythms, we show how predictive precision is degraded with decreasing sample points and increasing relative error. Additionally, we show how this method can be used for model discrimination by comparing the output identifiability of two candidate model structures to published literature data.

**Conclusions:**

Our method permits modellers of oscillatory systems to confidently show that a model’s dynamic characteristics follow directly from experimental data and model structure, relaxing assumptions on the particular parameters chosen. Ultimately, this work highlights the importance of continued collection of high-resolution data on gene and protein activity levels, as they allow the development of predictive mathematical models.

## Background

A cell’s behavior is governed by the dynamic and selective expression of its genes, in which each protein’s activity depends on a careful balance between transcription, translation, transport, and degradation rates. These rates, which change with environmental conditions and are often impossible to measure accurately in vivo or in vitro, determine the function of a regulatory pathway. While studying the roles of individual proteins can often provide some insight on how a particular function is achieved, this approach is limited in explaining complicated cellular phenomena at the scale of dozens to hundreds of interacting genes. With the aid of mathematical models, it is increasingly possible to create in silico realizations of genetic regulatory networks to examine their dynamic properties.

Essential to understanding how genetic circuits operate is connecting how inputs (i.e., environmental changes, extracellular signals) are processed to give the appropriate outputs (protein expression, cellular response). In some cases these quantities may be changes to oscillatory profiles: for example, seasonal changes in day length leading to flowering or hibernation. Models of genetic regulatory networks, often sets of ordinary differential equations (ODEs), contain many unknown parameters that must be estimated from experimental data [1]. Derivatives of the model output with respect to changes in input, known as local sensitivities, are frequently validated experimentally or used to predict potential targets for pharmaceuticals [2]. Since sensitivities can change drastically with respect to the particular parameter values chosen, the confidence associated with parameter and sensitivity values is an important consideration in model analysis and design.

Practical identifiability analysis is concerned with calculating confidence intervals in parameter estimates resulting from uncertainty in experimental data [3]. Several techniques for such an analysis currently exist, and are commonly used in analyzing biological models [4-6]. In one method, the inverse of the Fisher information matrix is used to provide estimates of the variance in each parameter. However, since this method assumes a linearized model, the resulting symmetric normal distributions for each parameter do not accurately reflect the mapping of nonlinear models [7]. In the bootstrap method, distributions in parameter estimates are found through optimum fits to repeated physical or in silico measurements. While accurate in finding the true nonlinear confidence intervals, this approach requires efficient and robust parameter estimation convergence.

Many systems biology models focus on describing interesting dynamic features from interlocked regulatory mechanisms. Limit cycle oscillations are common features in many biological networks, ranging from cell cycle control to cyclic firing of cardiac cells and circadian rhythms [8]. In periodic systems, the behavior (and existence) of limit cycle oscillations is a discontinuous function of the parameters, complicating parameter estimation. Optimal values are traditionally found through trial-and-error type approaches [9,10] or genetic algorithm search strategies [11], both of which are not amenable to bootstrap methods. Additionally, since the solutions are oscillatory, additional care must be taken in the calculation of the first-order sensitivity values. Here we calculate the sensitivity of the oscillatory period to parameter perturbation, a biologically relevant quantity that is often measured experimentally [12]. Due to these complications, rigorous identifiability analyses of these models are typically not performed.

In this study, a bootstrap uncertainty analysis appropriate for oscillatory biological models is developed and applied to a previously published model of circadian rhythms [13]. Circadian rhythms are near 24-hour endogenous oscillations in physiological processes found in many organisms, coordinated through transcription-translation networks with inherent time-delayed negative feedback [14-16]. In mammals, expression of circadian E box genes Period (Per) and Cryptochrome (Cry1 and Cry2) leads to elevated levels of their protein products, PER and CRY. The formation of a heterodimeric complex allows PER and CRY proteins enter the nucleus and subsequently suppress E-box mediated transcription, resulting in rhythmic gene expression. These networks serve as an excellent example of a functional genetic circuit, able to process subtle environmental cues while remaining robust to temperature variations and evolutionary disturbances. Accurate limit cycle models must capture not only the correct time-dependent dynamics, but also the correct input-output response. For circadian rhythms, high-throughput microarrays have provided high-resolution time-series data of gene expression levels [17]. Additionally, knockdown experiments using RNA interference technology (siRNA) and small molecule modulators have resulted in a wealth of data on the dynamic responses to changes in key rates [13,18-20]. This data, together with qualitative knowledge of the underlying network structure, permits the use and verification of a suitable uncertainty analysis.

To enable a bootstrap approach, we employ an efficient parameter estimation routine optimized for limit cycle models. Motivated by the increasing availability of high-resolution time-series measurements, we use an approach similar to multiple shooting, in which a nonlinear and discontinuous parameter estimation problem is transformed into a high-dimensional yet local optimization and solved via nonlinear programming [21]. Since the desired shape of the limit cycle solution is known a priori, a relatively accurate initial guess for the parameters and trajectories can be found. By using multiple sets of in silico data of varying quality, we illustrate how error in experimental results is propagated to uncertainty in parameter sensitivity. Lower quality data - with either higher error or fewer sampling points - result in wider distributions of limit cycles and less identifiable responses. These results can be used in a priori experimental design, finding the minimum sampling points needed for an estimated experimental error to enable accurate modeling. Additionally, we show using literature data how this method can be used to discriminate between candidate model structures, revealing which one yields the highest predictive confidence.

## Results and discussion

Mechanistic models of biological processes are often posed as nonlinear, time-invariant systems of ordinary differential equations (ODEs) [9-11], of the form:

(1) $$\frac{dx}{\text{dt}}=f(x(t),p)$$

in which the vector of state variables **x**(*t*) describe the time-dependent activity of important species (i.e., mRNA, proteins, or metabolites), the parameters **p** are the kinetic rate constants, and the vector function **f**(**x**(*t*),**p**) contains the transcription, translation, transport, and degradation rate laws of the gene regulatory network. In modeling rhythmic phenomena, we typically seek models and parameter values that display limit cycle oscillations - where for the solution approaches a non-trivial periodic trajectory:

(2) $$\underset{t\to \infty }{\text{lim}}x(t)=x(t+T).$$

Here the period of oscillation is the smallest *T*>0 in which the equality (2) holds. Limit cycle oscillations are independent of the system’s initial values **x**(0), and are instead determined completely by the parameters **p**.

Experimental values for **p** are rarely available. Given time-series experimental measurements ${\hat{x}}_{i}(t_{j})$ for each state variable in a limit cycle system, we find optimal parameters **p**^⋆^ such that the error between the experimental measurements and the simulated limit cycle is minimized [22]:

(3) $$p^{⋆}:=\arg\underset{p}{\text{min}}\overset{\text{states}}{\underset{i}{\sum }}\overset{\text{data}}{\underset{j}{\sum }}\frac{{\left({\hat{x}}_{i}(t_{j})-x_{i}(t_{j},p)\right)}^{2}}{\sigma_{\text{ij}}^{2}}.$$

Here *σ*_*ij*_ is the standard deviation associated with the measured mean of state *i* at time *j*. Using the data points ${\hat{x}}_{i}(t_{j})$ to generate a suitable initial guess, parameter estimation may proceed via a nonlinear programming approach (see Methods, Additional file 1). In this work, we assume that all states are measured to demonstrate how initial guesses can be generated directly from the input data. However, for systems with unmeasured states, initial guesses for the trajectory and parameter values can be provided by another approach, such as a global optimization routine. A bootstrap method was implemented by repeatedly sampling input data distributions to calculate a population of optimal parameter fits.

After finding optimal parameter fits, we used the models to predict how perturbations change systems dynamics by performing a first order sensitivity analysis. Since adjustments to periodic systems in response to inputs are often manifested through temporary changes in oscillatory period, relative period sensitivities,

(4) $$\frac{\partial \ln T}{\partial \ln p}$$

were calculated due to their independence of parameter magnitude [12,23,24]. Relative period sensitivities were integrated into the bootstrap method by calculating appropriate sensitivities for each estimated parameter set.

**Figure 1:**
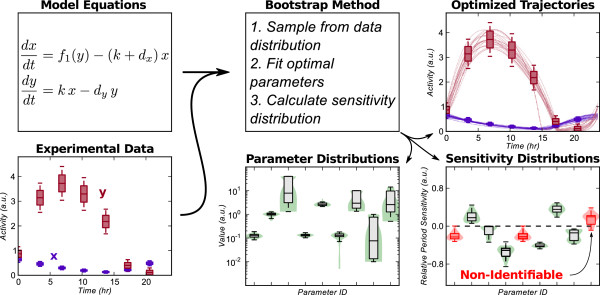
**Parameter estimation and bootstrap methods flowchart.** The demonstrated method calculates confidence intervals in the sensitivity of limit cycle models. An oscillatory model and experimental (or simulated) data are inputs to the bootstrap method. Unique data sets are then used to calculate optimum limit cycle trajectories. The resulting distribution in sensitivities highlight whether a particular response is identifiable (i.e., consistent across the majority of bootstrap trials).

Of particular importance in determining the reliability of a model prediction is whether an output response maintains a consistent direction despite noise in measurement data. We therefore define a sensitivity value to be practically identifiable for given input data if 95% of the distribution maintains a consistent sign, similar to definitions for parameter identifiability used in previous studies [7,25]. An overview of the method is shown in Figure 1.

### Effect of data quality on predictive confidence

**Figure 2:**
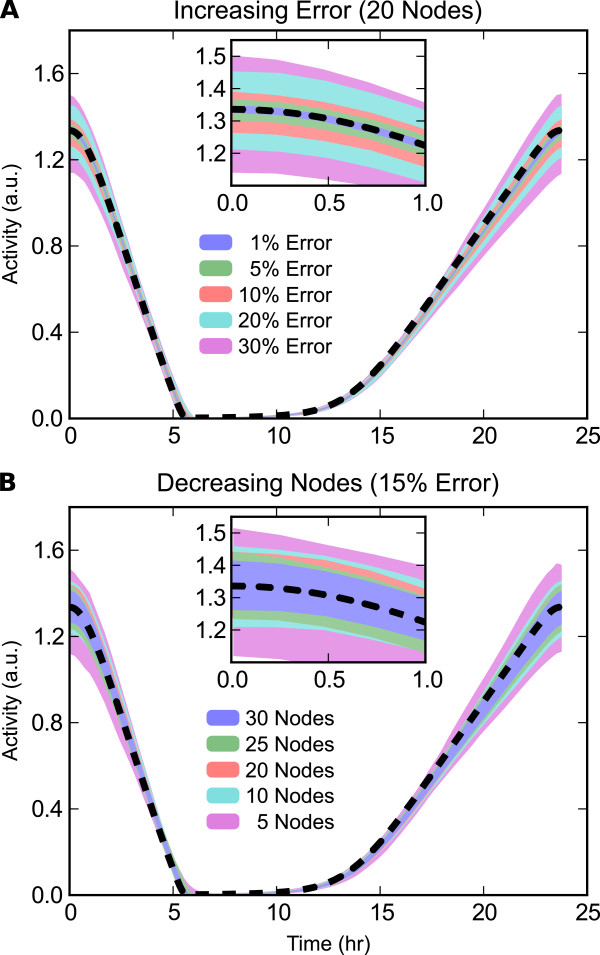
**Time-course profiles of the state trajectories for Per mRNA. (A)** Increasing relative error, *ξ*, with M=20. Possible state variable values are shown as shaded regions, obtained by filling between the 5 ^th^ and 95 ^th^ percentile for values at each time for 2000 independent parameter estimations. Increasing *ξ* results in larger deviations from the original model trajectory, shown as a dashed black line. **(B)** Decreasing number of measurement points, M, each with *ξ*=0.15. Higher M results in trajectories closer to the true trajectory.

We first analyze the degree to which uncertainty in input data is propagated to uncertainty in output predictions. To achieve this, we generate in silico data from a previously published model of circadian rhythms, using relative error *ξ* to generate normally distributed data ( $\sigma_{\text{ij}}\;=\;\xi \;{\hat{x}}_{i}(t_{j})$) at each of M sampling points. As expected, solution trajectories drifted further from the nominal limit cycle for higher values of error, *ξ*, or lower sampling density, M, (Figure 2). However, the overall shape of the oscillatory profiles remained relatively similar, even for rather high *ξ* or low M.

**Figure 3:**
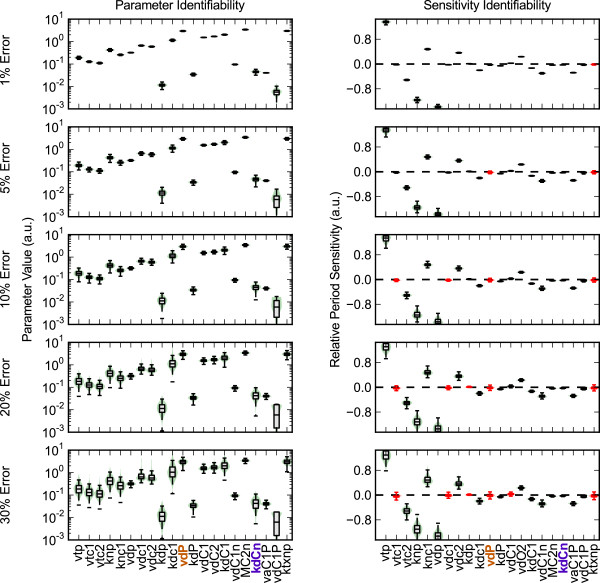
**Parameter and sensitivity identifiability for increasing error.** Increasing *ξ* results in a corresponding decrease in the confidence of the parameter and sensitivity estimates. Violin plots of the parameter values (left) and relative period sensitivities (right) show the distribution of values from each parameter estimation. In the plots, a box plot is superimposed above a kernel density plot to convey the distribution of values. The whiskers used extend to the most extreme data point within 1.5x the inner quartile range. Sensitivities in which the 5 ^th^ and 95 ^th^ percentile values span the x-axis are deemed non-identifiable (red), as the model’s response direction can not be accurately estimated. Higher *ξ* also results in wider parameter distributions.

**Figure 4:**
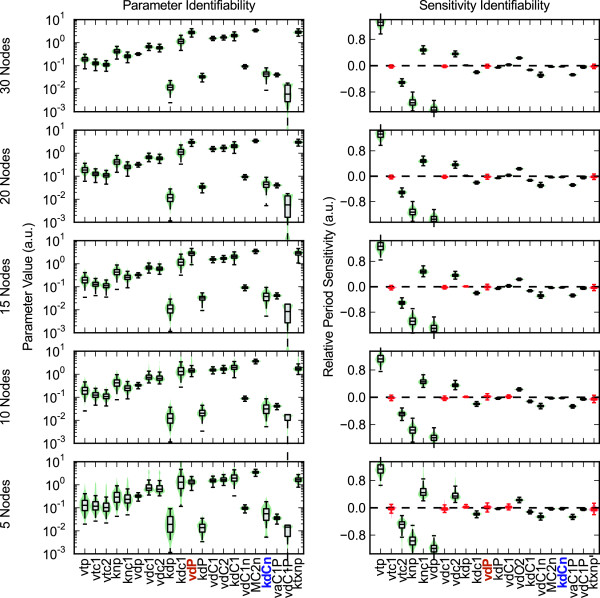
**Effect of high-resolution sampling on identifiability.** Lower values of M result in less constrained parameter and sensitivity values. Similar to Figure 3, violin plots of the parameters (left) and sensitivities (right) show the distribution from each parameter estimation for decreasing M. These results highlight the importance of high-resolution time sampling in generating sensitivity information for oscillatory models.

Figure 3 shows violin plots of the probability distribution for each parameter set and corresponding sensitivity evaluation for increasing *ξ*, while Figure 4 shows similar plots for decreasing M. Interestingly, there is little correlation between the identifiability of a parameter and its corresponding sensitivity value. For example, vdP, the maximum degradation rate of Per mRNA, shows a very tight clustering about its nominal parameter value, while the sensitivity of this parameter loses identifiability for even small values of *ξ*. Conversely, KdCn, the Michealis-Menten constant associated with the degradation of nuclear CRY, shows large variations in possible parameter values. However, the period sensitivity of KdCn, despite lying close to the x-axis, remains identifiable, indicating a robust prediction. These results reveal which model responses are constrained by the structure and dynamics of the limit cycle oscillations, and which are dependent on the particular parameterization chosen.

Sensitivities that are experimentally distinguishable from zero are the most important for validation. Calculating a typical experimental value for a relative period sensitivity helps to calibrate which sensitivities might be verified experimentally. Referring to a recent RNA interference screen, periods changes of approximately 1 hour (5%) can be reliably measured using luminescence recordings [18]. Assuming an increase in the corresponding mRNA degradation parameter value of 50%, this translates to a relative period sensitivity of 0.1. Thus, many of the identifiable values shown in Figures 3– 4 fall within the experimentally measurable range.

### Application to literature data for model discrimination

**Figure 5:**
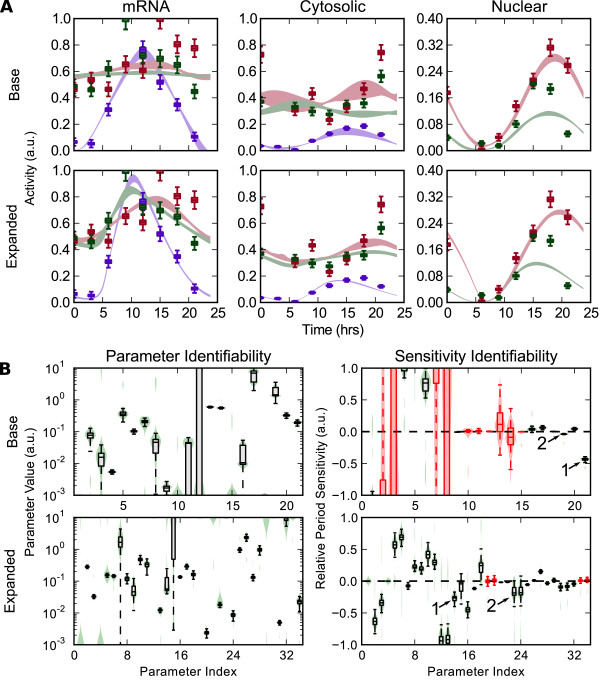
**Identifiability comparison of two model structures. (A)** Bootstrap parameter estimations on two model structures using literature time-series data with estimated errors (box plots). Resulting regions of model trajectories are shaded between the 5 ^th^ and 95 ^th^ percentile. Per species are shown in purple, Cry1 in red, and Cry2 in green. While both models were able to approximately reproduce the same dynamic response, the expanded model was better able to capture differences between the Cry1 and Cry2 profiles. **(B)** Parameter and sensitivity identifiability for the base and expanded models. Violin plots show the parameter and sensitivity distributions, with unidentifiable sensitivities (90% confidence level) highlighted in red. Despite containing more parameters, the expanded model shows better parameter identifiability and higher confidence in its predicted sensitivities. The PER translation rate ( 1) and PER-CRY association rate ( 2) sensitivities are consistent across model equations and are highlighted.

We next apply the method to literature time-course data for core clock components [26]. When modeling a genetic regulatory network, many candidate model equations are often considered. We show that a bootstrap uncertainty analysis can also be useful in discriminating between potential model structures based on predictive confidence. Here two variations of the same model are fit, see Additional file 2. The first model (Figure 5, base) was originally optimized using a genetic algorithm approach, and thus contains a minimal number of parameters to reduce optimization complexity. The second model considered (Figure 5, expanded) contains independent parameters for each rate expression, increasing the number of parameters from 23 to 35.

The literature data used consisted of 7-8 concentration time points across a 24 hour period. Confidence intervals in the data were not available, so an optimistic 3% relative and 0.5% absolute error was assumed for each data point ( $\sigma_{\text{ij}}=0.03\;{\hat{x}}_{i}(t_{j})+0.005\;\max({\hat{x}}_{i})$). Figure 5A shows the resulting time-series profiles for bootstrap estimations of each model. While additional kinetic parameters are typically thought to lower the predictive confidence of a model (the ‘curse of dimensionality’), the expanded model is able to better capture the oscillatory profiles with lower variability between solutions. Parameter and sensitivity distributions, Figure 5B, similarly show how the expanded model parameterization is able to generate more confident predictions in model response. Since the resulting sensitivity identifiability for both models was relatively poor, we highlight sensitivities which pass a 90% confidence level threshold. These results thus indicate higher-resolution data on circadian components would help in conferring confidence to model predictions.

Two sensitivities, the PER translation rate (Figure 5B, 1) and the PER-CRY association rate (2), had high confidence and consistent direction in both the base and expanded parameterization - suggesting that the predicted values are robust to slight changes in both parameter value and model structure. Since a biological system can be modeled using many different combinations of kinetic assumptions, such a technique will likely prove useful in finding consistent predictions which are robust to slight differences in model equations.

## Conclusions

Increasingly, mathematical models are being used to study biological systems where traditional experiments would prove infeasible. For example, in the search for drug targets, thousands of possible combinatorial perturbations can be quickly scanned for therapeutic effects using in silico modeling. This is especially useful in oscillatory systems with long periods, such as circadian rhythms, where a perturbed in vitro or in vivo system must be measured for multiple days before changes can be reliably determined.

However, since errors in model responses can arise from either incorrect structure or measurement noise, our confidence in in silico predictions is limited. Here we have developed a bootstrap approach suitable for periodic systems, and extended it to include uncertainty in predicted responses. With this method, errors due to local parameter effects can be identified, even in models with complicated dynamics. Furthermore, by considering multiple variations in model assumptions, we have demonstrated that a clearer result of trustworthy model predictions can be found.

Since this method takes advantage of time-series data to generate a strong initial guess for an otherwise difficult parameter estimation, it requires high-resolution data on the concentrations of all species in the model. In many biological systems, such data is only available for the activity levels of certain well-studied species. However, the continued development of high-throughput genomic and proteomic techniques promise to deliver time-series data for a much larger network of components. With expanding datasets, these methods will likely prove useful for the quantitative evaluation of uncertainty in larger biological models.

## Methods

### Generation of data for bootstrap methods

For each run, two thousand simulated measurements, ${\hat{x}}_{i}(t_{j})$, were generated from the true data, ${\tilde{x}}_{i}(t_{j})$, using a normal distribution with $\mu ={\tilde{x}}_{i}(t_{j})$ and $\sigma_{\text{ij}}=\xi \;{\tilde{x}}_{i}(t_{j})+\eta \;\underset{j}{\max}{\tilde{x}}_{i}(t_{j})$, in which *ξ* is the relative and *η* is the absolute error. Each simulated data set was then used to find a unique optimum parameter set, **p**^⋆^. Data sets that failed to converge, or reached a steady state solution (in which periodic sensitivities are undefined), were discarded from further analysis.

For the in silico data of varying quality used in Figures 2, 3- 4, we used the known limit cycle **x**(*t*) to generate data points ${\hat{x}}_{i}(t_{j})$ at each of M sampling points. The effect of increasing error and decreasing number sampling points were tested independently:

$$\begin{array}{lll} \xi & =\{.01,.05,.10,.20,.30\};\;M=20\; & \;\\ M & =\{30,20,15,10,5\};\;\;\;\xi =0.15\; & \; \end{array}$$

Since standard deviations in the data distributions were also used as optimization weights, a small amount of absolute error (*η*=0.001) was added to ensure errors in small values did not dominate the cost function.

### Collocation methods and sensitivity analysis

In this work, the estimation of the unknown kinetic parameters is accomplished via nonlinear programming (NLP) [21]. In this method, we divide the limit cycle trajectory into N finite elements, and approximate each with a K degree Lagrange interpolating polynomial, **x**(*t*). The minimization of (3) proceeds by changing the state variable and parameter values, ensuring both periodic continuity and system dynamics. The number of variables of the NLP problem is therefore (N)(K+1)(NEQ)+NP, where NEQ is the number of state variables and NP is the number of parameters. In the model considered in this study, with N=20, K=5, NEQ=8, and NP=21, the number of variables is 981. However, because suitable initial guesses can be found for both the state variable and parameter variables (see Figure 1), relatively efficient convergence can be achieved. Detailed information on the algorithms used are presented in Additional file 1.

### Calculation times

Each parameter estimation took approximately 4 seconds on a 2.53GHz processor, with the subsequent limit cycle solution integration and sensitivity calculation taking approximately 0.5 seconds. Due to the parallel nature of the 2000 trials, computation times were alleviated by distributing the tasks onto a cluster of 160 compute nodes.

### Software

The numerical implementation of the nonlinear programming optimizations was accomplished using IPOPT [27]. The CasADi computer algebra package [28] was used to provide an interface to the IPOPT numerical libraries and supply derivatives to the cost and equality function calls through automatic differentiation.

Other libraries used were the SUNDIALS [29] packages CVODES for ODE integration and KINSOL for the Newton iterations involved in the solution of the limit cycle. Integration of the sensitivity equations was performed by using the staggered-direct method from the CVODES integrator.

## Competing interests

The authors declared they have no competing interests.

## Authors’ contributions

PSJ and FJD designed the experiments and wrote the manuscript. PSJ implemented the method and ran all experiments. All authors read and approved the final manuscript.

## Supplementary Material

Additional file 1 **Supplemental methods.** Additional details on analytical and numerical methods used in the study, including collocation methods, generating initial values, and first order sensitivity analysis. Click here for file

Additional file 2 **Mathematical models.** Model equations and parameter values for the two models of circadian rhythms. Click here for file
