# Supplementary material for: Estimating confidence intervals in predicted responses for oscillatory biological models
Source: BMC Syst Biol. 2013 Jul 29;7:71. doi: 10.1186/1752-0509-7-71 (PMC3733791; doi:10.1186/1752-0509-7-71)
Supplement: Additional file 1 — Supplemental methods. Additional details on analytical and numerical methods used in the study, including collocation methods, generating initial values, and first order sensitivity analysis. [file 1752-0509-7-71-S1.pdf]

Supplemental Text 1

Collocation Methods

In this work, the estimation of the unknown kinetic parameters is accomplished via nonlinear programming. The method is summarized here, taken primarily from [1]. In this method, we divide the limit cycle trajectory $\boldsymbol{x}(t, \boldsymbol{p})$ into $\mathcal{N}$ finite elements of length h, and approximate each with a $\mathcal{K}$ degree Lagrange interpolating polynomial, $\boldsymbol{x}_i^{\mathcal{K}}(t)$, using an internal time $\tau \in [0, 1]$. For finite element i:

$$
\begin{aligned}
t &= h(i + \tau) \\
\ell_j(\tau) &= \prod_{k=0,\neq j}^{\mathcal{K}} \frac{\tau - \tau_k}{\tau_j - \tau_k} \\
\boldsymbol{x}_i^{\mathcal{K}}(\tau) &= \sum_{j=0}^{\mathcal{K}} \ell_j(\tau)\ \boldsymbol{x}_{ij}.
\end{aligned} \tag{1}
$$

We also ensure that the interpolating polynomial matches system dynamics at each collocation point, τ_k, by setting

$$
\begin{aligned}
\sum_{j=0}^{\mathcal{K}} \boldsymbol{x}_{ij} \frac{d\ell_j(\tau_k)}{d\tau} &= h\boldsymbol{f}(\boldsymbol{x}_{ij}, \boldsymbol{p}) \\
\text{for} \quad k &= 1, \ldots, \mathcal{K}.
\end{aligned} \tag{2}
$$

Additionally, the interpolating polynomials for each finite element must form a continuous function, so the following continuity constraints are imposed:

$$
\begin{aligned}
\boldsymbol{x}_{i+1,0} &= \sum_{j=0}^{\mathcal{K}} \ell_j(1)\ \boldsymbol{x}_{i,j} \\
\text{for} \quad i &= 1, \ldots, \mathcal{N} - 1.
\end{aligned} \tag{3}
$$

Periodic conditions are imposed by setting the beginning of the first element equal to the end of the final element:

$$
\boldsymbol{x}_{0,0} = \sum_{j=0}^{\mathcal{K}} \ell_j(1)\ \boldsymbol{x}_{\mathcal{N},j} \tag{4}
$$

The τ_k values are chosen for optimal accuracy, here we use Gauss-Radau roots so that the resulting method has stiff decay [1]. With $\mathcal{K} = 5$:

$$
\tau = \{0.000, 0.057, 0.277, 0.584, 0.860, 1.000\}
$$

The interpolating polynomials can now be compared to the experimental data. For each measured value, $\hat{\boldsymbol{x}}(t)$, the corresponding simulated values $\boldsymbol{x}^{\mathcal{K}}(t)$ can be interpolated from $\boldsymbol{x}_{ij}$:

$$
\boldsymbol{x}^{\mathcal{K}}(t) = \sum_{j=0}^{\mathcal{K}} \ell_j(\tau)\ \boldsymbol{x}_{ij} \tag{5}
$$

where i and τ are selected for the appropriate finite element and sampling time ($t = h(i + \tau)$, $\tau \in [0, 1)$). For $\mathcal{M}$ measurements, the objective function $\Phi(\boldsymbol{x}, \boldsymbol{p})$ is thus:

$$
\Phi(\boldsymbol{x}, \boldsymbol{p}) = \sum_{n}^{\mathcal{M}} \left(\frac{\boldsymbol{x}^{\mathcal{K}}(t_n) - \hat{\boldsymbol{x}}(t_n)}{\boldsymbol{\sigma}_n} \right)^2 \tag{6}
$$

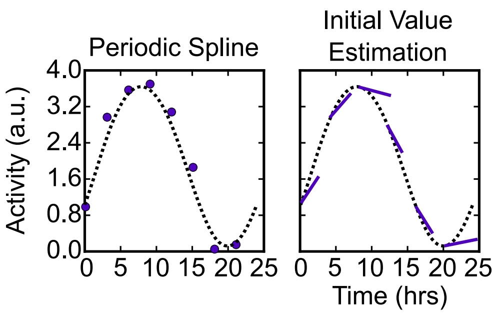

Figure S1. Parameter Estimation and Bootstrap Methods Summary.
Data points for each state are interpolated using a periodic spline to find starting values (left). The spline is differentiated to find the slope at each measurement point, which is used to find approximate initial values for the parameters (center). More data points thus improves the quality of the initial guess, and provides tighter control on the shape of the limit cycle. The optimization algorithm proceeds by fitting degree $\mathcal{K}$ interpolating polynomials to the data points, while enforcing continuity and dynamic constraints (right).

where $\boldsymbol{\sigma}_n$ is the measurement error associated with measurement n. Since $\boldsymbol{x}$ and $\boldsymbol{\sigma}$ are vectors, the division in 6 must be performed element-wise. This cost function was taken from a similar multiple-shooting approach to parameter estimation [2].

Since the cost function (6) and equality constraints (2, 3, and 4) now satisfy continuity and differentiability requirements [3], parameter estimation can now be accomplished via constrained nonlinear programming (NLP) instead of a global search strategy. The solution is subject to variable bounds:

$$\begin{aligned} \boldsymbol{x}_{LB} &\leq \boldsymbol{x} \leq \boldsymbol{x}_{UB} \\ \boldsymbol{p}_{LB} &\leq \boldsymbol{p} \leq \boldsymbol{p}_{UB} \end{aligned} \tag{7}$$

The numerical implementation is accomplished using IPOPT [4], using the `MA57` [5] linear solver. The CasADi computer algebra package [6] was used to provide an interface to the IPOPT numerical libraries and supply derivatives to the cost and equality function calls through automatic differentiation.

Generating Initial Values

Solution of the NLP described in section requires a suitable initial guess for the optimal state profiles, $\boldsymbol{x}^\star$, and kinetic parameters, $\boldsymbol{p}^\star$. To find approximate values for these variables, a smoothed periodic B-spline, $\tilde{\boldsymbol{x}}$, is found using experimental data for each state variable using SciPy's interpolate module [7] (Figure S1). Initial values for x_{ij} are obtained by evaluating this spline at each τ_k for each finite element.

$$\begin{aligned} & \boldsymbol{x}^\star_{ij} \approx \tilde{\boldsymbol{x}}(t_{ij}) \\ \text{where} \quad & t_{ij} = h(i-1+\tau_j) \\ \text{for} \quad & i = \{1,\ldots,\mathcal{N}\},\ j = \{1,\ldots,\mathcal{K}\} \end{aligned} \tag{8}$$

Since

$$\frac{d\tilde{\boldsymbol{x}}}{dt} \approx \boldsymbol{f}(\tilde{\boldsymbol{x}}, \boldsymbol{p}), \tag{9}$$

approximate values for $\boldsymbol{p}$ can be obtained by solving the simpler unconstrained NLP,

$$\min_{\boldsymbol{p}} \sum_{i}^{\mathcal{N}} \sum_{j}^{\mathcal{K}} \left(\frac{d\tilde{\boldsymbol{x}}(t_{ij})}{dt} - \boldsymbol{f}(\tilde{\boldsymbol{x}}(t_{ij}), \boldsymbol{p}) \right)^2 \tag{10}$$

in which t_{ij} is the same as in Equation 8 and the bounds on $\boldsymbol{p}$ are the same as in Equation 7.

First Order Sensitivity Analysis

After determining an optimal parameter set for the given experimental data, relevant first order sensitivity coefficients for oscillatory models are found using the procedure from [8], summarized here.

First, initial conditions and oscillatory period are verified by minimizing the norm of the vector:

$$\min_{\boldsymbol{x}(0),T} \left| \begin{array}{c} \boldsymbol{x}(T) - \boldsymbol{x}(0) \\ \dot{x}_0(0) \end{array} \right| \tag{11}$$

where $\dot{x}_0(0)$ denotes the time-derivative of the first state variable, evaluated at $t = 0$. By minimizing the derivative of the first state variable along with difference between final and initial values, we ensure the method converges to an identifiable point on the limit cycle. This boundary value problem is solved using Newton's method, employing the SUNDIALS packages CVODES for ODE integration and KINSOL for the Newton iterations [9].

Time-dependent parametric sensitivities,

$$\mathbf{S}(t) \equiv \frac{\partial \boldsymbol{x}(t)}{\partial \boldsymbol{p}},$$

are obtained by using the staggered-direct method from the CVODES integrator [10]. Sensitivities of the period, $\frac{\partial T}{\partial p}$, can be obtained directly from sensitivities integrated for one pass of the limit cycle (see [8] or [11] for further details) through a linear solve:

$$\begin{bmatrix} \mathbf{M} - \mathbf{I} & \dot{\boldsymbol{x}}(T) \\ \frac{\partial f_0}{\partial \boldsymbol{x}}(\boldsymbol{x}(0)) & 0 \end{bmatrix} \begin{bmatrix} \vdots \\ \frac{\partial T}{\partial \boldsymbol{p}} \end{bmatrix} = \begin{bmatrix} -\mathbf{S}(T) \\ -\frac{\partial \boldsymbol{f}}{\partial \boldsymbol{p}}(\boldsymbol{x}(0)) \end{bmatrix} \tag{12}$$

in which $\mathbf{M}$ is the Monodromy matrix, $\mathbf{I}$ is the identity matrix, and the unknown vector contains the relevant period sensitivities.

Since parameter values often span several orders of magnitude, an often more useful measure is the relative period sensitivity, which is independent of the magnitude of the period or parameter value.

$$\frac{\partial \ln T}{\partial \ln \boldsymbol{p}} = \frac{\boldsymbol{p}}{T} \frac{\partial T}{\partial \boldsymbol{p}} = \frac{\partial T}{T} \Big/ \frac{\partial \boldsymbol{p}}{\boldsymbol{p}} \tag{13}$$

Thus, a relative period sensitivity of 1 indicates that a 1% increase in the parameter value will result in a 1% increase in the period.

References

1. Biegler LT: *Nonlinear Programming: Concepts, Algorithms, and Applications to Chemical Processes*. Philidelphia: SIAM 2010.
2. Bock HG, Kostina E, Schloder JP: **Numerical methods for parameter estimation in nonlinear differential algebraic equations**. *GAMM-âĂŘMitteilungen* 2007, **408**(2):376 – 408.
3. Floudas CA: *Nonlinear and Mixed-Integer Optimization*. New York, New York, USA: Oxford University Press 1995.
4. Wachter A, Biegler LT: **On the implementation of an interior-point filter line-search algorithm for large-scale nonlinear programming**. *Math. Program.* 2006, **106**:25–57.
5. HSL: **A collection of Fortran codes for large scale scientific computation** 2011, [http://www.hsl.rl.ac.uk].
6. Andersson J, Åkesson J, Diehl M: **CasADi – A symbolic package for automatic differentiation and optimal control**. In *Recent Advances in Algorithmic Differentiation*, Lecture Notes in Computational Science and Engineering. Edited by Forth S, Hovland P, Phipps E, Utke J, Walther A, Berlin: Springer 2012.
7. Jones E, Oliphant T, Peterson P, et al.: **SciPy: Open source scientific tools for Python** 2001–, [http://www.scipy.org/].
8. Wilkins AK, Tidor B, White J, Barton PI: **Sensitivity Analysis for Oscillating Dynamical Systems**. *SIAM J. Sci. Comput.* 2009, **31**(4):2706–2732.
9. Hindmarsh AC, Brown PN, Grant KE, Lee SL, Serban R, Shumaker DANE, Woodward CS: **SUNDIALS: Suite of Nonlinear and Differential / Algebraic Equation Solvers**. *ACM T. Math. Software* 2005, **31**(3):363–396.
10. Serban R, Hindmarsh AC: **CVODES: the Sensitivity-Enabled ODE Solver in SUNDIALS**. In *Proceedings of IDETC/CIE 2005*, Long Beach, CA 2005:1–13.
11. Kramer M, Rabitz H, Calo J: **Sensitivity analysis of oscillatory systems**. *Appl. Math. Modell.* 1984, **8**(5):328–350.
