# Supplementary material for: Estimating confidence intervals in predicted responses for oscillatory biological models
Source: BMC Syst Biol. 2013 Jul 29;7:71. doi: 10.1186/1752-0509-7-71 (PMC3733791; doi:10.1186/1752-0509-7-71)
Supplement: Additional file 2 — Mathematical models. Model equations and parameter values for the two models of circadian rhythms. [file 1752-0509-7-71-S2.pdf]

Supplemental Text 2

Table 1: Model Equations for the Base Model. Lower case letters (p: *Per*, c1: *Cry1*, c2: *Cry2*) are mRNA state variables. Uppercase letters (P: PER, C1: CRY1, C2: CRY2) are the free (cytosolic) proteins. C1N: CRY1 and C2N: CRY2 are the nuclear proteins.

$$\frac{d\mathbf{p}}{dt} = \frac{\mathsf{vtp}}{\mathsf{knp} + (\mathbf{C1N} + \mathbf{C2N})^3} - \frac{\mathsf{vdp}\ \mathbf{p}}{\mathsf{kdp} + \mathbf{p}} \tag{1}$$

$$\frac{d\mathbf{c1}}{dt} = \frac{\mathsf{vtc1}}{\mathsf{knc1} + (\mathbf{C1N} + \mathbf{C2N})^3} - \frac{\mathsf{vdc1}\ \mathbf{c1}}{\mathsf{kdc1} + \mathbf{c1}} \tag{2}$$

$$\frac{d\mathbf{c2}}{dt} = \frac{\mathsf{vtc2}}{\mathsf{knc1} + (\mathbf{C1N} + \mathbf{C2N})^3} - \frac{\mathsf{vdc2}\ \mathbf{c2}}{\mathsf{kdc1} + \mathbf{c2}} \tag{3}$$

$$\begin{aligned}\frac{d\mathbf{P}}{dt} &= \mathsf{vtxnp}\ \mathbf{p} - \frac{\mathsf{vdP}\ \mathbf{P}}{\mathsf{kdP} + \mathbf{P}} - \mathsf{vaC1N}\ \mathbf{P}\ \mathbf{C1} + \mathsf{vdC1N}\ \mathbf{C1N} \\ &\quad - \mathsf{vaC1N}\ \mathbf{P}\ \mathbf{C2} + \mathsf{vdC1N}\ \mathbf{C2N}\end{aligned} \tag{4}$$

$$\frac{d\mathbf{C1}}{dt} = \mathsf{vtxnc1}\ \mathbf{c1} - \frac{\mathsf{vdC1}\ \mathbf{C1}}{\mathsf{kdC1} + \mathbf{C1}} - \mathsf{vaC1N}\ \mathbf{P}\ \mathbf{C1} + \mathsf{vdC1N}\ \mathbf{C1N} \tag{5}$$

$$\frac{d\mathbf{C2}}{dt} = \mathsf{vtxnc2}\ \mathbf{c2} - \frac{\mathsf{vdC2}\ \mathbf{C2}}{\mathsf{kdC1} + \mathbf{C2}} - \mathsf{vaC1N}\ \mathbf{P}\ \mathbf{C2} + \mathsf{vdC1N}\ \mathbf{C2N} \tag{6}$$

$$\frac{d\mathbf{C1N}}{dt} = -\frac{\mathsf{vdC1n}\ \mathbf{C1N}}{\mathsf{kdCn} + \mathbf{C1N} + \mathbf{C2N}} + \mathsf{vaC1N}\ \mathbf{P}\ \mathbf{C1} - \mathsf{vdC1N}\ \mathbf{C1N} \tag{7}$$

$$\frac{d\mathbf{C2N}}{dt} = -\frac{(\mathsf{vdC1n}\ \mathsf{MC2n})\ \mathbf{C2N}}{\mathsf{kdCn} + \mathbf{C2N} + \mathbf{C1N}} + \mathsf{vaC1N}\ \mathbf{P}\ \mathbf{C2} - \mathsf{vdC1N}\ \mathbf{C2N} \tag{8}$$

Table 2: Parameter Sets for Base model. Values and descriptions for model described in table 1. Original parameters found through genetic algorithm optimization, with translation rates (parameters 21-23) added after optimization. Refit parameters are obtained from the optimal fit to error-free data from the estimations shown in figure 5 (main text).

	Parameter	Description	Original	Refit
1	vtp	*Per* Transcription rate	1.946×10^{-01}	1.000×10^{-03}
2	vtc1	*Cry1* Transcription rate	1.306×10^{-01}	1.000×10^{-03}
3	vtc2	*Cry1* Transcription rate	1.135×10^{-01}	1.016×10^{-03}
4	knp	*Per* Repression constant	4.255×10^{-01}	1.000×10^{-03}
5	knc1	*Cry1/2* Repression constant	2.595×10^{-01}	1.173×10^{-02}
6	vdp	*Per* Max degradation rate	3.263×10^{-01}	4.606×10^{-01}
7	vdc1	*Cry1* Max degradation rate	6.761×10^{-01}	3.375×10^{-02}
8	vdc2	*Cry2* Max degradation rate	6.079×10^{-01}	3.428×10^{-02}
9	kdp	*Per* Degradation constant	1.148×10^{-02}	1.223
10	kdc1	*Cry1/2* Degradation constant	1.149	1.000×10^{-03}
11	vdP	Max PERc degradation rate	2.970	1.000×10^{-03}
12	kdP	PERc degradation constant	3.382×10^{-02}	1.000×10^{01}
13	vdC1	Max CRY1c degradation rate	1.523	2.849×10^{-01}
14	vdC2	Max CRY2c degradation rate	1.686	1.705×10^{-01}
15	kdC1	CRYc degradation constant	2.017	1.000×10^{-03}
16	vdC1n	CRYn degradation rate	1.012×10^{-01}	4.362×10^{-02}
17	MC2n	CRY2n degradation multiplier	3.318	9.829×10^{-01}
18	kdCn	CRYn degradation constant	5.263×10^{-02}	1.000×10^{-03}
19	vaC1N	CRYn association rate	4.063×10^{-02}	6.530×10^{-01}
20	vdC1N	CRYn dissociation rate	1.755×10^{-03}	1.000×10^{-03}
21	vtxnp	PER translation rate	3.000	1.065×10^{-01}
22	vtxnc1	CRY1 translation rate	1.000	4.729×10^{-01}
23	vtxnc2	CRY2 translation rate	1.000	3.020×10^{-01}

Table 3: Model Equations for the Expanded Model. These equations have similar reaction stoichiometry to those in table 1, but with more parametric degrees of freedom. This model showed better time-series performance than the more constrained model when fit to time-series data.

$$\frac{d\mathbf{p}}{dt} = \frac{\mathsf{Vm1}}{1 + \mathsf{Vm1}\left(\frac{\mathbf{C1N}+\mathbf{C2N}\ \mathsf{M1}}{\mathsf{Ki1}}\right)^3} - \frac{\mathsf{k1}\ \mathbf{p}}{1 + \frac{\mathbf{p}}{\mathsf{Km1}}} \quad (1)$$

$$\frac{d\mathbf{c1}}{dt} = \frac{\mathsf{Vm2}}{1 + \mathsf{Vm2}\left(\frac{\mathbf{C1N}+\mathbf{C2N}\ \mathsf{M2}}{\mathsf{Ki2}}\right)^3} - \frac{\mathsf{k2}\ \mathbf{c1}}{1 + \frac{\mathbf{c1}}{\mathsf{Km2}}} \quad (2)$$

$$\frac{d\mathbf{c2}}{dt} = \frac{\mathsf{Vm3}}{1 + \mathsf{Vm3}\left(\frac{\mathbf{C1N}+\mathbf{C2N}\ \mathsf{M3}}{\mathsf{Ki3}}\right)^3} - \frac{\mathsf{k3}\ \mathbf{c2}}{1 + \frac{\mathbf{c2}}{\mathsf{Km3}}} \quad (3)$$

$$\frac{d\mathbf{P}}{dt} = \mathsf{k4}\ \mathbf{p} + \mathsf{k12}\ \mathbf{C1N} + \mathsf{k13}\ \mathbf{C2N} - \frac{\mathsf{k7}\ \mathbf{P}}{1 + \frac{\mathbf{P}}{\mathsf{Km4}}} - \mathsf{k10}\ \mathbf{P}\ \mathbf{C1} - \mathsf{k11}\ \mathbf{P}\ \mathbf{C2} \quad (4)$$

$$\frac{d\mathbf{C1}}{dt} = \mathsf{k5}\ \mathbf{c1} + \mathsf{k12}\ \mathbf{C1N} - \frac{\mathsf{k8}\ \mathbf{C1}}{1 + \frac{\mathbf{C1}}{\mathsf{Km5}}} - \mathsf{k10}\ \mathbf{P}\ \mathbf{C1} \quad (5)$$

$$\frac{d\mathbf{C2}}{dt} = \mathsf{k6}\ \mathbf{c2} + \mathsf{k13}\ \mathbf{C2N} - \frac{\mathsf{k9}\ \mathbf{C2}}{1 + \frac{\mathbf{C2}}{\mathsf{Km6}}} - \mathsf{k11}\ \mathbf{P}\ \mathbf{C2} \quad (6)$$

$$\frac{d\mathbf{C1N}}{dt} = \mathsf{k10}\ \mathbf{P}\ \mathbf{C1} - \mathsf{k12}\ \mathbf{C1N} - \frac{\mathsf{k14}\ \mathbf{C1N}}{1 + \frac{\mathbf{C1N}+\mathbf{C2N}\ \mathsf{M4}}{\mathsf{Km7}}} \quad (7)$$

$$\frac{d\mathbf{C2N}}{dt} = \mathsf{k11}\ \mathbf{P}\ \mathbf{C2} - \mathsf{k13}\ \mathbf{C2N} - \frac{\mathsf{k15}\ \mathbf{C2N}}{1 + \frac{\mathbf{C1N}+\mathbf{C2N}\ \mathsf{M5}}{\mathsf{Km8}}} \quad (8)$$

Table 4: Parameter Set for Expanded Model. Parameters for model described in table 3, fit to time-series data via nonlinear programming.

	Parameter	Description	Value
1	M1	*Per*/CRY2 activity coefficient	3.632
2	Vm1	*Per* transcription rate	9.957×10^{-01}
3	Ki1	*Per*/CRY inhibition coefficient	1.054×10^{-01}
4	M2	*Cry1*/CRY2 activity coefficient	1.000×10^{-03}
5	Vm2	*Cry1* transcription rate	2.262×10^{-01}
6	Ki2	*Cry1*/CRY inhibition coefficient	2.049×10^{-01}
7	M3	*Cry2*/CRY2 activity coefficient	1.000×10^{01}
8	Vm3	*Cry2* transcription rate	1.850×10^{-01}
9	Ki3	*Cry2*/CRY inhibition coefficient	1.427×10^{-01}
10	k1	*Per* degradation rate	2.920×10^{-01}
11	Km1	*Per* degradation self-inhibition	6.609×10^{-01}
12	k2	*Cry1* degradation rate	1.000×10^{01}
13	Km2	*Cry1* degradation self-inhibition	1.823×10^{-02}
14	k3	*Cry2* degradation rate	4.711×10^{-02}
15	Km3	*Cry2* degradation self-inhibition	1.000×10^{01}
16	k4	*Per* translation rate	1.132×10^{-01}
17	k5	*Cry1* translation rate	3.409×10^{-01}
18	k6	*Cry2* translation rate	1.961×10^{-01}
19	k7	PER degradation rate	1.000×10^{01}
20	Km4	PER degradation self-inhibition	1.911×10^{-03}
21	k8	CRY1 degradation rate	1.000×10^{01}
22	Km5	CRY1 degradation self-inhibition	2.077×10^{-02}
23	k9	CRY2 degradation rate	1.000×10^{01}
24	Km6	CRY2 degradation self-inhibition	1.180×10^{-02}
25	k10	C1N association rate	5.022×10^{-01}
26	k11	C2N association rate	1.035
27	k12	C1N dissociation rate	1.000×10^{-03}
28	k13	C2N dissociation rate	2.070×10^{-01}
29	M4	CRY1n/CRY2n activity coefficient	1.000×10^{01}
30	k14	CRY1N degradation rate	1.000×10^{01}
31	Km7	CRY1n degradation inhibition	5.206×10^{-03}
32	M5	CRY2n/CRY2n activity coefficient	1.000×10^{01}
33	k15	CRY2n degradation rate	1.000×10^{01}
34	Km8	CRY2n degradation inhibition	1.829×10^{-02}
